# The Year of the Mammoth

**DOI:** 10.1371/journal.pbio.0040078

**Published:** 2006-02-07

**Authors:** Alan Cooper

## Abstract

Three recently published studies that independently sequenced the mitochondrial genomes of the long extinct mammoth are examined here, along with the state-of-the-art technologies used to study ancient DNA.

Mammoth mitochondrial (mt) genomes are apparently on a similar schedule to London buses—you wait for ages and then suddenly three come along at once. Within the past six weeks, three studies [1–3] have independently determined all, or most, of the mammoth mt genome sequence, some 16,800 base pairs (bp). Encouragingly, the partial sequence was a byproduct of a study that generated some 13 million bp of mammoth genomic DNA using a new, massively parallel sequencing approach. The very divergent methods used in these three studies also neatly represent the past, present, and future of ancient DNA (aDNA) research.

aDNA methods provide an opportunity to characterise the genetic composition of species and populations in the past, and to actually observe evolutionary change through real time. Such a record has great potential to reveal the processes that have generated the diversity and distribution of taxa in our modern environment, and to examine phenomena such as speciation, domestication, morphological evolution, and the impacts of major environmental changes. aDNA data also provide an important opportunity to test our ability to accurately reconstruct evolutionary history via the fossil record or via extrapolation from the genetic data of modern species. Unfortunately, the potential of aDNA remains largely untapped because research has been severely limited by the technical difficulties of retrieving and studying the trace amounts of highly fragmented DNA that survive in ancient specimens.

## Background: Two Decades of aDNA Research

The first aDNA studies, in the mid-1980s, found that DNA degradation occurred rapidly after death, and only tiny amounts of short fragments (100–200 bp) could be retrieved from mummified specimens, even after just a few years at normal temperatures [4]. The aDNA molecules were both fragmented and damaged, and cross-linked to proteins, and surviving endogenous sequences were dwarfed by large amounts of microbial and fungal DNA, presumably introduced postmortem. As a result, aDNA was an extremely poor template for the existing bacterial cloning approaches.

In the late 1980s, the enormous amplifying power of the polymerase chain reaction (PCR) stimulated a rapid increase in aDNA research since it became possible to amplify and characterise even a few surviving copies of a short genetic sequence, despite the presence of overwhelming amounts of other DNA. Bone and teeth were quickly found to be far better sources of aDNA than soft tissues [5,6], and this meant museums suddenly became recognised as vast storehouses of preserved genetic information. PCR studies commonly targeted mt genes because their high copy number (approximately, 1,000 mt genomes are present per cell versus just the two chromosomal copies of any unique nuclear sequence) favoured survival in degraded ancient tissues. While encoding a small number of genes (37 in mammals), mt sequences were commonly used in phylogenetic and phylogeographic studies of living taxa, due to their maternal inheritance and relatively rapid evolution, allowing easy integration of the aDNA data [7–9]. PCR and biochemical analyses soon revealed why aDNA molecules were so difficult to work with—hydrolytic attacks broke the backbone of DNA strands while oxidatively damaged sites blocked polymerase action, and condensation reactions cross-linked proteins and DNA [4,10–13]. The most common form of damage detected (the deamination of cytosine residues) caused a sequence substitution (a C→T or G→A change), and was estimated to effect almost 2% of the cytosines in some specimens [12].

The amplifying power of PCR came at a cost, however, as only a single molecule of undamaged modern DNA (including previously amplified aDNA sequences) could out-compete the damaged aDNA and contaminate the reaction. Ironically, this problem was made much worse by the enormous number of molecules generated by PCR itself, since a successful reaction can produce roughly 10^14^ copies of the target sequence. In contrast, an average ancient specimen contains only about 10^3^–10^6^ copies of an mt sequence per gram [14,15]. To avoid being swamped by the resulting concentration differential (eight orders of magnitude), aDNA research had to be isolated in dedicated clean rooms, far from modern biology laboratories. Even when extreme measures were used to avoid laboratory contamination, the ancient specimens themselves were found to be permeated with modern human DNA introduced by handling and washing during archaeological excavation or museum storage [13,16,17]. This problem has effectively constrained studies of human aDNA since the contaminating sequences are often similar, or potentially even identical, to the authentic DNA.

## A Need for Better Methods

Unfortunately, aDNA research has not kept pace with the phenomenal growth in other areas of molecular biology since the early 1990s. The standard method has remained essentially unaltered: samples are digested with proteolytic enzymes, DNA is isolated with organic solvents (or silica), and a small aliquot of the extracted DNA is used to amplify a specific short sequence via PCR. It has been possible to stitch together multiple short fragments to generate long sequences, and, indeed, complete mt genome sequences were determined for the extinct moa, a giant ratite bird from New Zealand, in this way [15,18]. However, the standard approach means that every PCR amplification consumes an aliquot of the valuable and limited aDNA extract, although, because only a single short genetic target is amplified, nearly all of the DNA in the aliquot is ignored. Consequently, highly damaged, low-concentration DNA extracts can rapidly be consumed in the generation of even relatively short sequences (e.g., the original Feldhofer Neandertal). Further destruction of valuable specimens can be hard to justify, and, really, an intrinsically less wasteful approach is required, where more of the DNA in each aliquot is amplified during the PCR.

## Mammoth DNA

The phylogenetic relationship of the mammoth to living African and Asian elephants remains unresolved, with previous morphological and molecular studies equivocal or conflicting [3]. The speciation events between the three species are thought to have occurred in rapid succession around 6 million years ago in Africa, leaving few signals of the series of events. Further phylogenetic resolution would require long sequences from the mammoth, and the three recent studies have all used remains preserved in Siberian permafrost deposits to do just this.

Of the three studies, Rogaev et al. [3] used traditional aDNA methods on the oldest specimen. DNA was extracted from multiple 100- to 400-mg muscle samples of a 32,000-year-old mammoth leg from Chukotka in separate laboratories in Russia and the United States. Many PCR amplifications were then used to generate 35 fragments of 500–600 bp, which together spanned the entire mt genome*(DQ316067)*. The results from each laboratory, and from longer fragments of up to 1,600 bp, matched completely, apart from a few substitutions attributable to cytosine deamination and a short hypervariable sequence within the control region. Complete mt genome sequences of both living elephants were also generated*(DQ316068, DQ316069)*. Phylogenetic analyses suggested a closer relationship between the mammoth and the Asian elephant, to the exclusion of the African elephant, but confirm that the speciation events occurred rapidly—perhaps around 4 million years ago.

Krause et al. [1] reached a similar conclusion using a powerful variant of the PCR method known as multiplexing. Multiplexed PCRs differ from standard PCRs by simultaneously amplifying multiple genetic targets instead of just one. Consequently, much more of the DNA aliquot is actually used as a template for amplification. Like Rogaev et al., Krause et al. used the mt genome sequences of the modern elephants to design PCR primers to amplify the entire sequence. In this case, 46 fragments of 290–580 bp were targeted for simultaneous amplification using DNA extracted from a 200-mg bone sample of a 12,000-year-old mammoth from Yakutia. Each fragment was subsequently reamplified from the multiplex PCRs, and the resulting sequences were compared with each other and with those from independent experiments in two other laboratories. Sequence variation due to putative deaminated cytosine residues was called using a “best of three” rule, and the results were assembled into a complete mt genome sequence*(NC_007596)*. Intriguingly, while the two mammoth genomes were very similar, comparisons with the Asian elephant revealed that the much older Chukotka sequence actually had 25% less independent polymorphisms, suggesting that it was better preserved and that fewer deaminated cytosines may have resulted in a more accurate sequence [3]. Krause et al. [1] also found that the African elephant diverged first, but that the Asian and mammoth lines diverged only 440,000 years later—about 5.6 million years ago. The contrasting date estimates of the two studies are caused by different approaches to dealing with evolutionary rate variation and the problematically distant outgroups (the dugong and rock hyrax, which diverged from elephantids at least 65 million years ago). Ironically, the solution will probably be to sequence the mt genome of another extinct elephantid—the mastodon, which diverged as recently as 23 million years ago.

Importantly, the Krause et al. study indicates that the entire mt genomes of extinct species can potentially be determined with just the same amount of DNA as is used in a standard single-locus PCR. A close living relative is required for primer design, and very short DNA fragments dramatically increase the number of fragments required, but the method should work well with most permafrost megafauna.

If the Krause et al. study [1] represents the current state of play in aDNA research, the third study represents the future. Poinar et al. [2] exploit a recently developed, massively parallel sequencing system developed by 454 Life Sciences [19] to determine over 13 million bp of mammoth nuclear and mt sequences in a single experiment (NCBI SID 131303). A large amount (0.73 µg) of DNA was extracted from an exceptionally well-preserved 28,000-year-old mandible from Taimyr, and purified and concentrated using standard methods. Short primer sequences were then enzymatically “linked” to the ends of all the DNA fragments present, including contaminating microbial sequences, to facilitate nonspecific amplification. (Unfortunately, this critical process is relatively inefficient with most aDNA extracts). One of the many innovations of the 454 method is that the amplification step is performed within an emulsion, where millions of different fragments are amplified in separate droplets without interacting with one another. This avoids the laborious and expensive large-scale bacterial cloning steps used in standard genomic sequencing, and is a huge improvement in efficiency and cost. The amplified fragments are also sequenced in parallel, in picolitre wells on a fibre optic slide, using a pyrosequencing method where light is released when bases are incorporated into a growing DNA molecule. A CCD camera records the emitted light, and software translates the signals from each well into sequence data and assembles the short fragments into longer stretches. Technical constraints currently limit individual pyrosequencing reads to around 100–150 bp, but the already fragmented aDNA is well suited to this limitation. Using this method, Poinar et al. were able to generate over 300,000 short sequences (average 95 bp), of which 45% aligned to a single position within the elephant genome. In the process, around 11,000 bp of the mt genome was determined as well. Poinar et al. suggest that the entire nuclear genome could be characterised for this specimen, although it is not clear how long stretches of highly repetitive regions could be negotiated with such fragmented DNA.

## The Future

While these studies provide an exciting opportunity to dramatically increase the amount of genetic information available from ancient material, it is important to remember that the mammoths are exceptionally well-preserved ancient specimens containing very large amounts of DNA. In nonfrozen conditions, DNA is preserved in far smaller amounts and fragment sizes, and with much higher microbial DNA content, as shown by a recent study of DNA from a 40,000-year-old European cave bear that contained only 1%–6% carnivore sequences [20]. Sequence modifications caused by deaminated cytosines will remain a significant problem for genomic studies, as shown by the Krause et al. study, and will require many overlapping sequences for accurate characterisation.

The major requirement for aDNA research now appears to be a PCR-based method to amplify the trace amounts of aDNA from normal specimens up to the levels required for the linker-based 454 sequencing approach. This would have the associated advantage of creating libraries of amplified fragments for each specimen, removing the need for further specimen destruction and providing an almost infinite resource for future research.

This is an exciting time, as the opportunities provided by the new parallel sequencing system will allow researchers to contemplate large-scale studies of ancient genomes, and promise to finally release the full potential of aDNA to reveal evolution in action.

## Abbreviations

aDNA: ancient DNA
bp: base pair
mt: mitochondrial
PCR: polymerase chain reaction
